# S-Nitrosylation Proteome Profile of Peripheral Blood Mononuclear Cells in Human Heart Failure

**DOI:** 10.1155/2016/1384523

**Published:** 2016-08-18

**Authors:** Sue-jie Koo, Heidi M. Spratt, Kizhake V. Soman, Susan Stafford, Shivali Gupta, John R. Petersen, Maria P. Zago, Muge N. Kuyumcu-Martinez, Allan R. Brasier, John E. Wiktorowicz, Nisha Jain Garg

**Affiliations:** ^1^Department of Pathology, University of Texas Medical Branch (UTMB), Galveston, TX 77555, USA; ^2^Department Preventive Medicine and Community Health, UTMB, Galveston, TX 77555, USA; ^3^Institute for Translational Sciences, UTMB, Galveston, TX 77555, USA; ^4^Department of Biochemistry and Molecular Biology, Sealy Center of Molecular Medicine, UTMB, Galveston TX 77555, USA; ^5^Department of Microbiology and Immunology, UTMB, Galveston, TX 77555, USA; ^6^Instituto de Patología Experimental, CONICET-UNSa, 4400 Salta, Argentina; ^7^Department of Internal Medicine-Endocrinology, UTMB, Galveston, TX 77555, USA; ^8^Institute for Human Infections and Immunity, UTMB, Galveston, TX 77555, USA

## Abstract

Nitric oxide (NO) protects the heart against ischemic injury; however, NO- and superoxide-dependent S-nitrosylation (S-NO) of cysteines can affect function of target proteins and play a role in disease outcome. We employed 2D-GE with thiol-labeling FL-maleimide dye and MALDI-TOF MS/MS to capture the quantitative changes in abundance and S-NO proteome of HF patients (versus healthy controls, *n* = 30/group). We identified 93 differentially abundant (59-increased/34-decreased) and 111 S-NO-modified (63-increased/48-decreased) protein spots, respectively, in HF subjects (versus controls, fold-change | ≥1.5|, *p* ≤ 0.05). Ingenuity pathway analysis of proteome datasets suggested that the pathways involved in phagocytes' migration, free radical production, and cell death were activated and fatty acid metabolism was decreased in HF subjects. Multivariate adaptive regression splines modeling of datasets identified a panel of proteins that will provide >90% prediction success in classifying HF subjects. Proteomic profiling identified ATP-synthase, thrombospondin-1 (THBS1), and vinculin (VCL) as top differentially abundant and S-NO-modified proteins, and these proteins were verified by Western blotting and ELISA in different set of HF subjects. We conclude that differential abundance and S-NO modification of proteins serve as a mechanism in regulating cell viability and free radical production, and THBS1 and VCL evaluation will potentially be useful in the prediction of heart failure.

## 1. Introduction

Of the 57 million global deaths annually, 17.3 million (~30%) are due to cardiovascular diseases [1, 2]. Heart failure (HF) is a clinical syndrome that manifests as a consequence of the diverse factors including myocardial infarction, hypertension, cardiomyopathies, and atrial fibrillation. The intimate relationship of the micro- and macroenvironment with the cardiomyocytes results in cellular events that may be important to the initiation and propagation of HF, though a clear understanding of the molecular mechanisms underlying HF is not available. Some studies have identified HF-associated alterations in Ca^2+^ handling, energy metabolism, and contractile function in experimental models (reviewed in [3]); however, how these processes promote HF pathophysiology and if these processes are relevant to human HF remain unclear.

Nitric oxide (NO) protects the heart against ischemic injury, and NO-based therapy is part of the standard of care in patients with heart failure [4]. The classic view holds that NO acts primarily as a vasodilator; however, it is not known how NO protects the ischemic heart. There has been growing appreciation that endogenous nitrosylating compounds called S-nitrosothiols are involved in ischemic cardioprotection [5]. An alternative view is that NO present in conjunction with ROS results in the formation of peroxynitrite (H_2_O_2_ + NO_2_^−^ → ONOO^−^ + H_2_O) that is highly stable and soluble and can induce S-nitrosylation (S-NO) of cysteine residues on target proteins [6], potentially altering their function. Thus, protein S-NO modification may ameliorate cardiac injury or cause protein dysfunction.

Currently there are a limited number of clinically approved biomarkers available for the management of the entire spectrum of cardiovascular diseases [7]. These markers include serum cholesterol total/LDL, creatine kinase myocardial isoform (CK-MB), cardiac troponins (cTnI and cTnT), and brain natriuretic peptide (BNP) or its precursor N-terminus isoform (NT-proBNP). Of these only BNP/NT-proBNP has been validated for heart failure patients [8]. These biomarkers, though useful, provide a limited view of the disease status, and there is a need for the identification of new biomarkers of HF.

Peripheral blood mononuclear cells (i.e., PBMCs) carry the inherent genetic signature of the host and serve as an accessible and useful tissue capable of detecting and responding to stimuli, similar to what may be induced in the body [9–12]. Recent studies have distinguished the gene expression profile in peripheral blood leukocytes of stroke patients [13, 14]. Yet, proteomics, which incorporates the comprehensive characterization of all facets of protein biology, including the determination of protein localization, modifications, interactions, activities, and ultimately their function, is particularly well suited for advancing our understanding of complex disease mechanisms like HF. Importantly, the proteome is highly dynamic owing to a large range of protein expression and presence of a myriad of protein forms or “proteoforms” [15] that may arise from mutations, truncations, alternative splicing events, and the addition of posttranslational modifications (PTMs).

In this study, we aimed to identify the networks of proteins that are disturbed in abundance or posttranslational S-NO modifications and determine the evolution of chronic HF in human patients. Peripheral blood cells are shown to produce nitric oxide in various clinical situations, and a significant increase in the expression and activity of inducible nitric oxide synthase is noted in PBMCs of chronic heart failure patients [16]. We utilized saturation thiol-labeling maleimide dye that exhibits stable, specific, quantitative labeling of cysteine residues in conjunction with 2D-GE approach and mass spectrometry [17–19] for developing the PBMCs proteome of the normal healthy (NH) and HF subjects (*n* = 30/group). Importantly, our approach included steps that allowed simultaneous discovery of protein abundance and S-NO modifications for evaluating the PBMC's proteome [20–22]. This approach combines the resolving power of two-dimension gel electrophoresis (2D-GE) [23, 24] with the highly quantitative nature of saturation fluorescence [25] to permit S-NO and abundance change quantification with minimal processing steps that lead to major losses experienced by other S-NO technology (e.g., biotin-switch technique). Proteome datasets were submitted to modeling analysis for (a) identifying the potential pathways that were disturbed and (b) top molecules that were altered in abundance or S-NO levels in HF subjects. We have employed traditional ELISA, Western blotting, and biotin-switch assay for targeted analysis of a new batch of PBMC samples (*n* = 15/group) and verified the differential abundance and S-NO levels of THBS1 polypeptide and VCL in HF (versusNH) subjects. We discuss the molecular mechanisms that might be disturbed in progression of HF.

## 2. Materials and Methods

### 2.1. Ethics Statement for the Use of Human Samples

All procedures were approved by the Institutional Review Boards at the UTMB, Galveston. Samples were decoded and deidentified before they were utilized for research purposes. Blood samples (10 mL) were collected with K_3_EDTA (1.5-mg/mL blood). Subjects with a degree of systolic dysfunction (ejection fraction: ≤54%), left ventricular dilatation (end diastolic diameter ≥57 mm), and blood levels of NT-proBNP >1000 ng/L that reflects NYHA classification II-III of cardiac involvement were identified as those at risk of heart failure. Subjects with no signs of systolic dysfunction (ejection fraction: ≥55) or ventricular dilation and blood levels of NT-proBNP <400 ng/L were included as healthy controls. For the proteomic studies, blood samples were obtained from HF subjects (*n* = 30, 56% males, age range: 58–71 years, average: 62 years) and NH controls (*n* = 30, 60% males, age range: 28–65 years, average: 59.5 years).

### 2.2. PBMC Isolation, BODIPY Labeling, and 2D-GE

All chemicals and reagents were of molecular grade (>99.5% purity). Heparinized Vacutainer CPT Cell Preparation Tubes (Becton Dickinson, Franklin Lakes, NJ) containing <8 mL of whole blood samples were centrifuged following manufacturer's instruction. The PBMCs were harvested using a FICOLL*™* Hypaque*™* density gradient (GE Healthcare, Piscataway, NJ) and centrifuged again at 300 ×g for 10 min at room temperature to pellet the cells. The cell pellets consisting of 8–10-million PBMCs were washed with RPMI-1640 medium and stored at −80°C until analysis.

PBMC pellets from individual study subjects were lysed in 7 M urea, 2 M thiourea, 2% CHAPS, and 50 mM Tris (pH 7.5), containing benzonase nuclease (300-units/mL) [24]. Protein concentrations were determined with the Lowry method and cysteines (cysteic acid) determined by amino acid analysis (Model L8800, Hitachi High Technologies America, Pleasanton, CA) [18, 20]. Samples thus analyzed yielded ~200 pmol of Cys/*μ*g of protein. At least 92% of all human proteins contain at least one cysteine residue [26] and thus can be detected using the thiol-labeling maleimide dye [18, 20]. To detect the changes in abundance and S-NO modification levels, each sample was split into two fractions (each fraction containing 100 *μ*g of protein) [27]. Because some cysteine thiols are hyperreactive that can be oxidized under aerobic conditions in few minutes if without any protection [28], we immediately treated one fraction with copper chelator neocuproine (100 *µ*M for 1 h) that is shown to prevent S-NO reduction and stabilize S-NO during further processing of samples. The second protein fraction was incubated for 1 h with 6 mM ascorbate to ensure all cysteine residues were reduced and available for dye-binding. Both fractions were dialyzed against the urea buffer in the cold to remove ascorbate or to serve as a process control and then labeled with BODIPY® FL* N*-(2-aminoethyl) maleimide (BD from Life Technologies, Grand Island, NY) at 60-fold excess to cysteine that ensured sufficient dye was available to label all available cysteine residues [20], thereby ensuring reproducibility and accuracy. The mixtures were incubated for 2 h; then the reactions were stopped with a 10-fold molar excess of 2-mercaptoethanol (2-ME) over dye. All incubations were carried out at room temperature in the dark in 200 *μ*L reaction volume. As we utilized 100 *µ*g protein sample labeled with 60-fold molar excess dye over thiol all within a volume of 200 *µ*L, we used 6 *µ*mol/mL of BD per sample, and 60 *µ*mol/mL of 2-ME.

All BD-labeled, Asc^+^ and Asc^−^ PBMC lysates (100 *µ*g protein) were separated by 2-dimensional gel electrophoresis (2D-GE), employing an IPGphor multiple sample isoelectric focusing (IEF) device (GE Healthcare) in the first dimension and the Criterion Dodeca cell (Bio-Rad, Hercules, CA) in the second dimension [29, 30]. Sample aliquots were first loaded onto 11 cm dehydrated precast immobilized pH gradient (IPG) strips (pH range 3–11, from GE Healthcare) and rehydrated overnight. IEF was performed at 20°C with the following parameters: 50 V, 11 hours; 250 V, 1 hour; 500 V, 1 hour; 1,000 V, 1 hour; 8,000 V, 2 hours; 8,000 V, 48,000 V/hr. The IPG strips were then incubated in 10 mL of equilibration buffer (6 M urea, 2% sodium dodecyl sulfate (SDS), and 50 mM Tris-HCl, pH 8.8, 20% glycerol) for 30 minutes at 22°C with shaking [29, 30]. Electrophoresis was performed at 150 V for 2.25 hours, 4°C with precast 8–16% polyacrylamide gels in Tris-glycine-SDS buffer (25 mM Tris-HCl, 192 mM glycine, 0.1% SDS, pH 8.3) [29, 30].

### 2.3. Image Processing and Analysis

In total, 120 BODIPY-stained 2D-GE gels (2 gels with either Asc^+^ or Asc^−^ protein lysates per sample for the HF (*n* = 30) and NH (*n* = 30) subjects) were run by us. After electrophoresis, gels were fixed in 20% methanol, 7% acetic acid, and 10% acetonitrile for 1 h and washed with 20% ethanol and 10% acetonitrile to reduce background. The gels were imaged at 100 *µ*m resolution using the Typhoon Trio Variable Mode Imager (GE Healthcare) to quantify BD-labeled proteins (Ex_488 nm_/Em_520 nm_).

All gels were analyzed using the SameSpots*™* software. The current version of Totallab Ltd. SameSpots software (formerly Nonlinear Dynamics, Ltd., Newcastle, UK), unlike traditional analysis, does not rely on propagating and matching spots to an arbitrary reference. Instead, it relies on geometric correction of the scans themselves and projecting them all into the same reference space, performing pixel-to-pixel matching before spot detection. This approach ensures that spot boundaries are the same for all gels, eliminating errors that accumulate in the reference gel(s) as the number of gels within one experiment increases. The software selects one reference gel according to several criteria including quality and number of spots with the intent on selecting the gel that best represents all the gels. The reference gel containing the most common features was selected from the pool of gels. To ensure that maximum number of proteins were detected, the reference gel was stained with SyproRuby (from Life Technologies, Grand Island, NY) and scanned at Ex_488 nm_/Em_560 nm_ to ensure that all proteins (irrespective of presence or absence of cysteine residue) were detected. The exposure time for both dyes was adjusted to achieve a value of ~55,000–63,000 pixel intensity (16-bit saturation) from the most intense protein spots on the gel [29, 30].

For identifying the differential proteome, all the 2D gel images were assessed for quality control by SameSpots software and then aligned both manually and automatically against the reference gel. After manual and automated pixel-to-pixel alignment, spot boundaries were detected and the fluorescence intensity of each protein spot was normalized by using a bias factor calculated assuming most spots did not change across the experiment. The SyproRuby stained reference gel was used to define spot boundaries; however, the gel images taken under the BD-specific filters were used to obtain the quantitative spot data. This strategy ensured that spot numbers and outlines were identical across all gels in the experiment, eliminating problems with unmatched spots [30] as well as ensuring that the greatest number of protein spots and their spot volumes were accurately detected and quantified.

The detailed protocol for quantification of protein abundance and cysteinyl-S-nitrosylation by BD labeling is recently described by us [20]. Briefly, protein spot abundance ratios were calculated from normalized spot volumes from Asc^+^ HF sample versus the matched normal spot volumes (Δprotein abundance = Asc^+^ HF/Asc^+^ NH). Spot volumes were normalized for each sample using a software-calculated bias value assuming that the great majority of spot volumes did not change in abundance (log (abundance ratio) = 0). The scatter of the log (abundance ratios) for each spot in a gel (sample) is distributed around a mean value that represents the systematic factors that govern the experimental variation. Thus, a gain factor is calculated to adjust the mean spot ratios of a given gel to 0 (log (abundance ratio) = 0) and applied to each spot volume [23]. The S-NO modification levels were quantified by calculation of the ratio of fluorescence units from Asc^−^ aliquots (ΔS-NO = Asc^−^ HF/Asc^−^ NH). Finally, the ratio of ratios, that is, ΔS-NO/Δprotein abundance = [Asc^−^ HF/Asc^−^ NH]/[Asc^+^ HF/Asc^+^ NH], was calculated to obtain the change in S-NO levels normalized for protein abundance [20, 21]. As S-NO modification inhibits the Cys-BODIPY fluorescence; a negative RoR value would indicate an increase in S-NO level (and vice versa) in the sample.

For the purpose of selecting differentially abundant and S-NO-modified protein spots for mass spectrometry, normalized spot volumes were subjected to statistical analysis using in-built tools in Totallab SameSpots software. Spot volumes were log2 transformed and spot-wise standard deviation, arithmetic mean, and coefficient of variation (CoV) values of the standard abundance (and S-NO) were calculated for each spot [31]. Student's* t*-test with Welch's correction for unequal variances was used to test for differential protein abundance and S-NO level between NH controls and HF subjects. Benjamini-Hochberg multiple hypothesis testing correction was applied to account for the false discovery rate and significance was accepted at *p* < 0.05. The protein spots identified to be differentially abundant or differentially S-NO modified (fold change |≥1.5|, *p* < 0.05) in HF subjects were submitted for mass spectrometry identification.

### 2.4. Mass Spectrometry and Protein Identification

Selected 2D-GE spots that exhibited significant differential prevalence in HF group were picked robotically (ProPick II, Digilab, Ann Arbor, MI) [16]. Gel spots were incubated at 37°C for 30 min in 50 mM NH_4_HCO_3_, dehydrated twice for 5 min each in 100 *µ*L acetonitrile, and dried, and proteins were digested in-gel at 37°C overnight with 10 *µ*L of trypsin solution (1% trypsin in 25 mM ammonium bicarbonate). Peptide mixtures (1-*µ*L) obtained after tryptic digestion were purified with a ZipTip C_18_ column (Millipore) and reconstituted with 0.4% acetic acid. A 1 : 1 dilution of peptide solution with MALDI matrix solution (5 mg alpha-cyano-4-hydroxycinnamic acid per mL in 50% acetonitrile) was spotted on to the target plate and analyzed by matrix assisted laser desorption ionization-time of flight (MALDI-TOF) mass spectrometry (MS) using a MALDI-TOF/TOF ABI 4800 Proteomics Analyzer (AB Sciex, Foster City, CA). The Applied Biosystems software package included the 4000 Series Explorer (v.3.6 RC1) with Oracle Database Schema (v.3.19.0) and Data Version (3.80.0) to acquire and analyze MS and MS/MS spectral data. The instrument was operated in a positive ion reflectron mode with the focus mass set at 1700 Da (mass range: 850–3000 Da). For MS data, 1000–2000 laser shots were acquired and averaged from each protein spot. Automatic external calibration was performed by using a peptide mixture with the reference masses 904.468, 1296.685, 1570.677, and 2465.199. Following MALDI MS analysis, MALDI MS/MS was performed on several (5–10) abundant ions from each protein spot. A 1-kV positive ion MS/MS method was used to acquire data under postsource decay (PSD) conditions. The instrument precursor selection window was ±3 Da. Automatic external calibration was performed by using reference fragment masses 175.120, 480.257, 684.347, 1056.475, and 1441.635 (from precursor mass 1570.700) [32, 33].

AB Sciex GPS Explorer*™* (v.3.6) software was employed in conjunction with MASCOT (v.2.2.07) to search the UniProt human protein database (last accessed: June 7, 2015; 87,656 sequences 35,208,664 residues) by using both MS and MS/MS spectral data for protein identification [33]. Protein match probabilities were determined by using expectation values and/or MASCOT protein scores. The MS peak filtering included the following parameters: a mass range of 800 Da to 3000 Da, minimum S/N filter = 10, mass exclusion list tolerance = 0.5 Da, and mass exclusion list for some trypsin and keratin-containing compounds included masses (Da) 842.51, 870.45, 1045.56, 1179.60, 1277.71, 1475.79, and 2211.1. The MS/MS peak filtering included the following parameters: minimum S/N filter = 10, maximum missed cleavages = 1, fixed modification of carbamidomethyl (C), variable modifications due to oxidation (M), precursor tolerance = 0.2 Da, MS/MS fragment tolerance = 0.3 Da, mass = monoisotopic, and peptide charges = +1. The significance of a protein match, based on the peptide mass fingerprint (PMF) in the MS and the MS/MS data from several precursor ions, is presented as expectation values (*p* < 0.001).

Protein spots (|≥2| fold change) identified with low confidence by MALDI MS/MS (protein score <62) were submitted for analysis by LTQ OrbiTrap Velos (ThermoFisher, Waltham, MA).

### 2.5. Functional Analysis and Multivariate Adaptive Regression Splines (MARS) Modeling

The protein datasets were assessed by using ingenuity pathway analysis (IPA, Ingenuity Systems®). IPA retrieves biological information from the literature—such as gene name, subcellular location, tissue specificity, function, and association with disease—and then integrates the identified proteins into networks and signaling pathways with biological interpretation and significance [34]. An “*e*-value” was calculated by estimating the probability of a random set of proteins having a frequency of annotation for that term greater than the frequency obtained in the real set, and a significance threshold of 10^−3^ was used to identify significant molecular functions and biological processes [33]. With these parameters, we were able to highlight the most informative and significantly over-represented gene ontology terms in the dataset [35].

For MARS modeling, log2-transformed values of normalized spot volumes for all spots from 120 gels were exported from SameSpots in to Excel and analyzed by using R and SPSS ver.20 software. For modeling the disease state specific response, a stringent cut-off was applied; differentially abundant protein spots were first screened by *t*-test/Welch's correction and then Benjamini-Hochberg test was employed at *p* < 0.001 (|≥1.5| fold change). MARS was employed to model changes in multiple variables for distinguishing between infection and disease status [31]. To avoid overfitting the data, we employed two approaches: (1) 10-fold cross-validation (CV), allowing the same number of maximum basis functions as were the differentially abundant protein spots at *p* < 0.001 (with 1 max interaction term), and (2) testing/training approach in which 80% of the data was utilized for creating the model and the 20% of the remaining data was used to assess the fit of the model for testing dataset. The sensitivity and specificity of the identified models were validated by receiver operator characteristics (ROC) curves [31].

### 2.6. Enzyme-Linked Immunosorbent Assay (ELISA) and Biotin-Switch Assay

A new batch of PBMC samples from HF and NH subjects (*n* = 15 per group) were lysed by sonication in cold PBS and the protein concentrations were evaluated by the Bradford method (Bio-Rad). A sandwich ELISA kit was used to quantify the vinculin (VCL) abundance, following the manufacturer's instructions (Cloud-Clone Corp, Houston TX; sensitivity: ≥30-pg/mL). Briefly, PBMC lysates (5-*µ*g/100 *µ*L/well) were loaded onto 96-well plates precoated with VCL-specific antibody. After overnight incubation at 4°C, plates were aspirated and sequentially incubated with biotin-conjugated anti-VCL 2nd antibody (1 : 100 dilution), avidin-conjugated horseradish peroxidase (HRP) (1 : 100 dilution), and TMB substrate. The change in absorbance was measured at 450 nm by using a Spectramax 190 spectrophotometer (Molecular Devices, Sunnyvale, CA). The plates were washed between each reagent addition and a standard curve was prepared by using recombinant VCL protein (0–5000 pg/mL).

THBS1 abundance in PBMC lysates was quantified by using a sandwich ELISA kit (R&D Systems, Minneapolis, MN; sensitivity: ≥350-pg/mL). In brief, PBMC lysates (5 *µ*g/50 *µ*L/well) were loaded with 100 *μ*L of the provided assay diluent onto 96-well plates precoated with THBS1-specific monoclonal antibody and incubated overnight at 4°C. The plates were washed and incubated with HRP-conjugated THBS1 polyclonal antibody before addition of the TMB substrate. Absorbance was measured at 450 nm, and a standard curve was prepared by using 0–1000 ng/mL recombinant THBS1 protein.

The levels of S-NO modified VCL and THBS1 in PBMC lysates were determined by performing a biotin-switch assay followed by ELISA. Briefly, PBMC lysates were made as above, free SH (thiol) groups were blocked, protein S-NO bonds were present in the sample cleaved, and the newly formed SH groups were biotinylated using an S-Nitrosylated Protein Detection Assay Kit (Cayman Chemicals, Ann Arbor, MI) according to instructions provided by the manufacturer. The 96-well plates were coated for 2 h at room temperature with anti-VCL (Cloud-Clone Corp) or anti-THBS1 (R&D Systems) antibody (1 : 1000 dilution in Tris-buffered saline, TBS). Plates were then blocked for 2 h at room temperature with 5% BSA in TBS, washed three times with TBS, and incubated for overnight at 4°C with biotin-derivatized protein lysates (5 *µ*g/100 *µ*L/well). Plates were washed to remove the unbound proteins and then incubated for 30 minutes at room temperature with streptavidin-HRP conjugate (1 : 3000 dilution; BioLegend, San Diego, CA). Color was developed using the TMB substrate, and the change in absorbance reflecting the levels of biotin-bound S-NO modified VCL or THBS1 was measured by spectrophotometry.

### 2.7. Western Blotting

A 5 *µ*g aliquot of each protein sample was resolved on 10% acrylamide gels and wet-transferred to PVDF membranes by using a vertical Criterion Blotter (Bio-Rad). Membranes were blocked for 1 hour with 5% nonfat dry milk (Lab Scientific, Highlands, NJ) in 20 mM Tris Base (pH 7.4) containing 150 mM NaCl and 0.1% Tween-20 (TBST). All antibody dilutions were made in 3% bovine serum albumin (Fisher Scientific, Pittsburgh, PA) in TBST. Membranes were sequentially incubated overnight at 4°C with polyclonal rabbit anti-THBS1 antibody (0.5 *µ*g/mL dilution, Abcam, Cambridge, MA) and goat anti-rabbit HRP-conjugated secondary antibody (1 : 10,000 dilution, Southern Biotech, Birmingham, AL), and the signal was developed by using the Amersham*™* ECL Plus system (GE Healthcare). Images were visualized and digitized by using the ImageQuant system (GE Healthcare).

## 3. Results

### 3.1. 2D-GE/MALDI MS Identification of Changes in PBMC Proteome in Heart Failure

**Figure 1:**
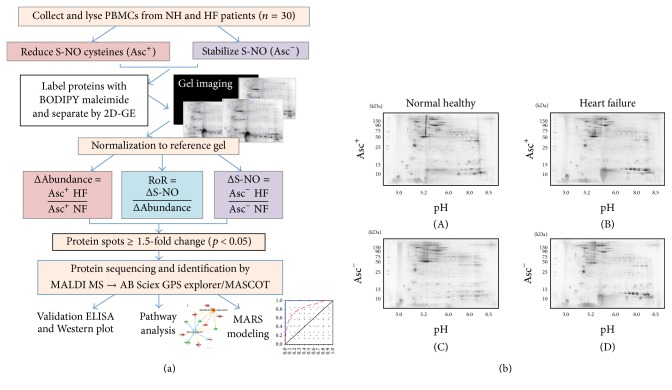
(a) Schematic work flow. PBMCs were obtained from heart failure subjects (HF, *n* = 30) and normal healthy (NH, *n* = 30) subjects. Each sample was divided into two fractions, and S-NO cysteines were reduced with ascorbate (Asc^+^) in one fraction and stabilized with neocuproine in 2nd fraction (Asc^−^). All fractions were labeled with BODIPY FL *N*-(2-aminoethyl) maleimide (binding to reduced cysteine) and resolved by 2-dimensional gel electrophoresis. Gel images were normalized against a reference gel. Ratiometric calculation of differential protein abundance from BODIPY-fluorescence units in Asc^+^ aliquots (normal versus experimental) was calculated for all the protein spots (Δprotein abundance = Asc^+^ HF/Asc^+^ NH). The S-NO modification levels were quantified by calculation of the ratio of fluorescence units from Asc^−^ aliquots (ΔS-NO = Asc^−^ HF/Asc^−^ NH). The ratio of ratios (RoR), that is, ΔS-NO/Δprotein abundance = [Asc^−^ HF/Asc^−^ NH]/[Asc^+^ HF/Asc^+^ NH], was calculated to obtain the change in S-NO levels normalized for protein abundance. The fold changes in abundance and S-NO-modification of the protein spots in all gels were log transformed and subjected to statistical analysis as described in Materials and Methods. Protein spots that changed in abundance or S-NO modification by |≥1.5-fold| at *p* < 0.05 were submitted to mass spectrometry analysis for protein identification. The protein datasets were analyzed by ingenuity pathway analysis and MARS modeling, and selected proteins were confirmed for differential abundance and S-NO modification levels by multiple assays. (b) Two-dimensional gel images of protein spots in PBMCs of heart failure (HF) subjects and normal healthy controls. BD-labeled PBMC lysates were separated in the 1st-dimension by isoelectric focusing on 11 cm nonlinear pH 3–11 immobilized pH gradient strips and in the 2nd-dimension by sodium dodecyl sulfate polyacrylamide gel electrophoresis (SDS-PAGE) on an 8–16% gradient gel. Gel images were obtained at 100 *µ*m resolution using the Typhoon Trio Variable Mode Imager (GE Healthcare) to quantify BD-labeled proteins (Ex_488 nm_/Em_520±15 nm_). Shown are representative gel images of Asc^+^ ((A) and (B)) and Asc^−^ ((C) and (D)) PBMCs from NH ((A) and (C)) controls and HF ((B) and (D)) subjects and approximate size (vertical) and pI (horizontal) ranges.

A schematic of work flow is presented in Figure 1(a). We employed a saturation fluorescence approach using BODIPY FL-maleimide (BD, dye to protein thiol ratio of >60 : 1) that specifically labels protein thiols to give an uncharged product with no nonspecific labeling [17, 18]. BD-labeled protein isoelectric points were unchanged and mobilities were identical to those in the unlabeled state. The Typhoon Trio Variable Mode Imager has a linear dynamic range of over four orders of magnitude and was capable of detecting 5 fmol of BD-labeled protein in a gel spot at a signal-to-noise ratio of 2 : 1 [17–19]. To ensure saturation labeling, protein extracts were analyzed for cysteine content and sufficient dye was added to achieve the desired excess of dye to thiol. This saturation fluorescence labeling method yielded high accuracy (>91%) in quantifying blinded protein samples [30] and, in a study that detected >1000 proteins across 6 experimental treatments, exhibited <9% CVs across triplicate runs.

The BD-labeled Asc^+^ and Asc^−^ PBMC lysates of NH controls (*n* = 30) and HF subjects (*n* = 30) were resolved by 2D-GE to obtain a disease-specific protein abundance and S-NO modification signature. Representative Asc^+^ and Asc^−^ gel images from each group are shown in Figure 1(b)((A)–(D)). All protein spots within the relative molecular sizes of 10 to 250 kDa after reduction and denaturation were detected. Some proteins may fall outside this size range and may have been missed; however, since all proteins were denatured and eventually reduced, we feel this is a minor limitation, no more so than the limited acetonitrile elution range and data dependent modes typically used in mass spec-centric approaches.

**Figure 3:**
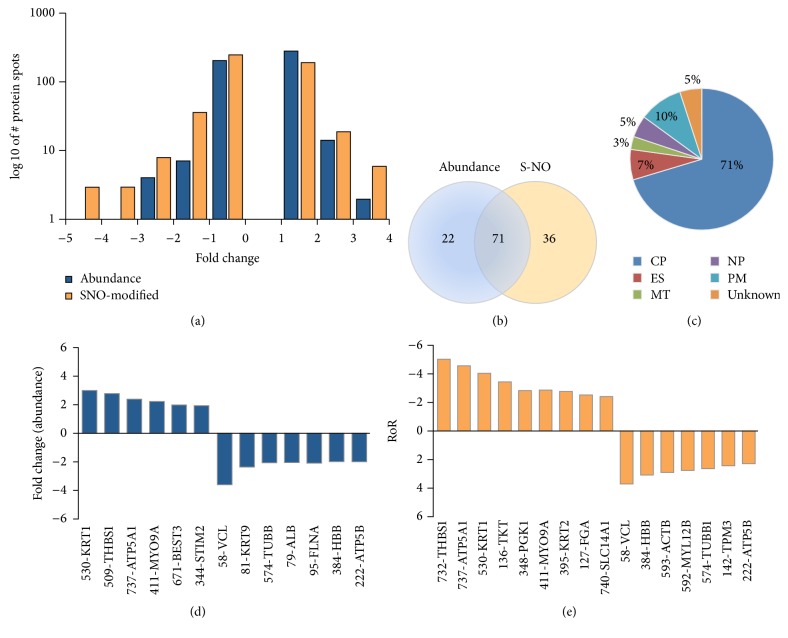
(a) Frequency of changes in abundance of protein spots in HF subjects. Shown is the frequency of protein spots that were changed in abundance or S-NO modification in HF subjects with respect to normal controls (*p* < 0.05). (b) Venn diagram. Shown is the number of protein spots that were increased in abundance and/or S-NO modification levels in HF subjects. (c) Classification of differentially expressed proteins from the proteomic analysis. Ontological classification of differentially regulated proteins in terms of cellular localization was performed by ingenuity pathway analysis. The compositions of the protein categories are presented as percentages of all individually identified proteins. CP: cytoplasmic, ES: extracellular/secreted, MT: mitochondrial, NP: nucleoplasm, PM: plasma membrane. ((d) and (e)) Fold change in abundance (d) and S-NO modification (e) of top molecules identified to be of relevance in HF subjects. Ratio of ratio (RoR) is defined in legend of Figure 2. A negative RoR indicates increased S-NO modification, while a positive RoR indicates increased reduction of protein thiols.

We identified 93 differentially abundant protein spots (59 upregulated, 34 downregulated, and fold change: |≥1.5|, *p* < 0.05) and 111 differentially S-NO modified protein spots (63 low RoR, 48 high RoR, and fold change: |≥1.5|, *p* < 0.05) in HF (versus NH) subjects with high confidence (Table 1). The changes in abundance and Cys-S-NO modification frequency of the protein spots in HF subjects ranged from 3.00-fold to −3.61-fold and −5.05-fold to 3.76-fold, respectively (Figure 3(a)). Further, 71 protein spots were changed in both abundance and Cys-S-NO levels, while 22 and 36 protein spots were uniquely changed in abundance or S-NO modification levels, respectively, in HF subjects (Figure 3(b)). The protein spots that were changed in abundance or in Cys-S-NO levels were predicted to be localized mostly in the cytoplasm (71%), with the rest distributed to the nucleus (5%), plasma membrane (10%), or extracellular space (7%) (Figure 3(c)). Top differentially expressed (Figure 3(d)) and S-NO-modified (Figure 3(e)) protein spots in HF subjects, which were identified by ingenuity pathway analysis (IPA), are presented. Note that KRT1, THBS1, ATP5A1, and MYO9A were increased in abundance as well as S-NO modification levels, while VCL, HBB, and ATP5B were decreased in abundance and S-NO modification levels in HF subjects.

### 3.2. Pathway Network Analysis of the Disease-Associated Proteome Signature

The protein abundance and S-NO modification datasets from HF versus NH controls (Table 1) were submitted to IPA to determine molecular and biological functions, as well as the important pathways and networks involved in HF risk. IPA analysis of the differentially abundant proteome dataset predicted a putative increase in platelet aggregation (*z* score: 1.432), phagocyte chemotaxis/migration (*z* score: 1.091, *p* value 3.84*E* − 09), and free radical production (*z* score: 1.491, *p* value 2.23*E* − 03) with a decline in leukocyte/neutrophil activation and fatty acid metabolism in HF development (Figures S1A and S1B in Supplementary Material available online at http://dx.doi.org/10.1155/2016/1384523). The molecular and cellular function annotation of the differentially abundant proteome dataset predicted an increase in cell death (*z* score: 1.989, 21 molecules, and *p* value 5.41*E* − 05) and a decline in cell survival and cell viability (*z* score: −0.809, 11 molecules, and *p* value 2.39*E* − 03) in HF subjects (Figure S2).

IPA analysis of the differential S-NO proteome dataset showed increased S-NO-modification of several molecules predicted to be involved in migration of phagocytes (Figure S3). However, increased S-NO-modification of other molecules was predicted to inhibit cell spreading and development of blood vessels in HF subjects (Figure S3). Likewise, we noted increased S-NO-modification of several molecules predicted to be involved in inhibition of apoptosis and activation of organismal death and free radical production in HF subjects (Figure S4). These data suggested that changes in abundance and S-NO modification serve as an important mechanism in regulating inflammation and cellular survival in HF.

### 3.3. MARS Modeling Identifies Proteins Predictive of HF

**Figure 4:**
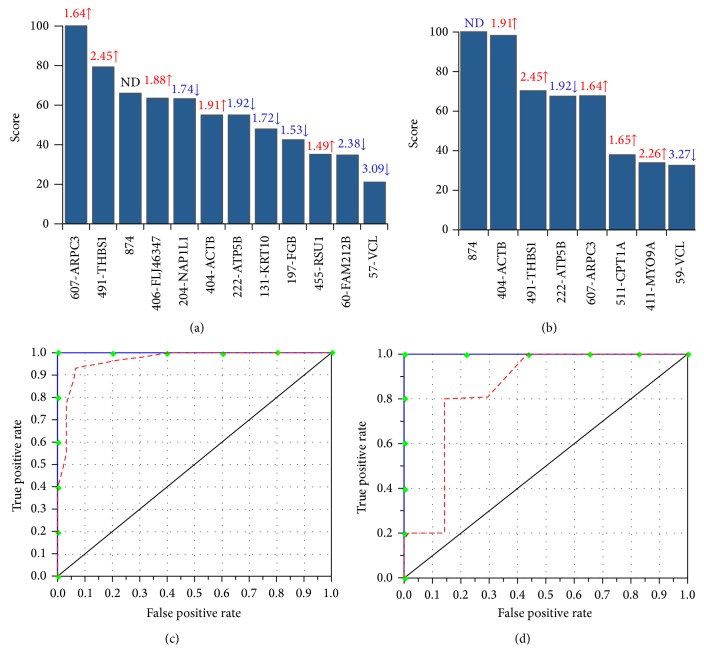
MARS analysis of differentially abundant protein spots in HF subjects. Inputs to the model were protein spots that were differentially abundant at *p* < 0.05 with B-H correction in HF (31 spots, *n* = 30) subjects with respect to NH controls (*n* = 30). We employed 10-fold cross-validation ((a) and (c)) and 80% testing/20% training ((c) and (d)) approaches to assess the fit of the model for testing dataset. Shown are the protein spots identified with high ranking (score >20) by CV (a) and 80/20 (b) approaches for creating the MARS model for classifying HF from NH subjects. Protein spots in panels (a) and (b) are identified as spot number-protein name, and fold changes (increase ↑, red; decrease ↓, blue) are plotted. The ROC curves show the prediction success of the CV (c) and 80/20 (d) models. Blue curves: training data (AUC/ROC: 1.00), red curve: testing data (AUC/ROC: 0.97 for CV and 0.857 for 80/20).

**Figure 5:**
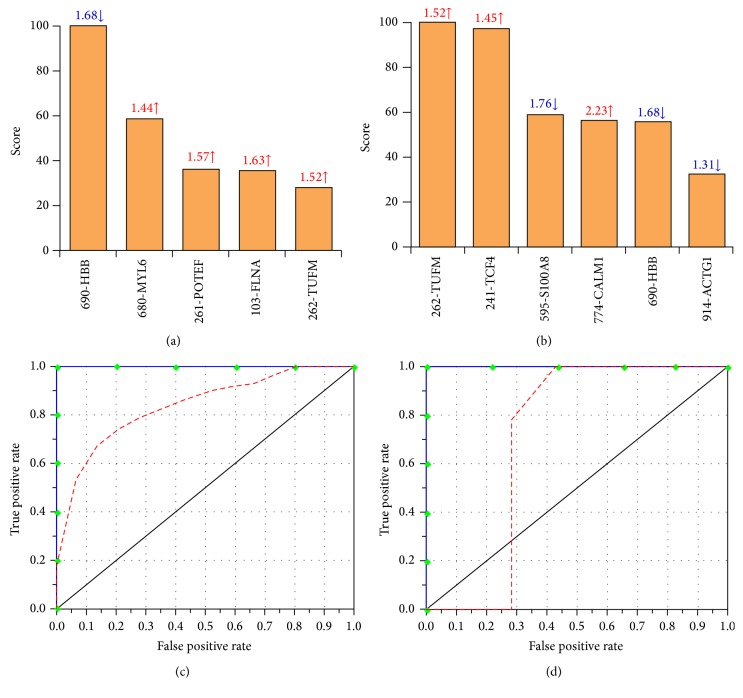
MARS analysis of differentially S-NO modified protein spots in HF subjects. Inputs to the model were protein spots that were differentially S-NO modified at *p* < 0.05 with B-H correction in HF (42 spots, *n* = 30) subjects with respect to NH controls (*n* = 30). We employed 10-fold cross-validation ((a) and (c)) and 80% testing/20% training ((c) and (d)) approaches to assess the fit of the model for testing dataset. Shown are the protein spots identified with high ranking (score >20) by CV (a) and 80/20 (b) approaches for creating the MARS model for classifying HF subjects from NH controls. Protein spots in panels (a) and (b) are identified as spot #-protein name, and RoR values (increase ↑, red; decrease ↓, blue) are plotted. The ROC curves show the prediction success of the CV (c) and 80/20 models (d). Blue curves: training data ((AUC/ROC: 1.00); red curve: testing data (AUC/ROC: 0.85 for CV and 0.714 for 80/20).

We performed MARS analysis on our proteome dataset to develop a classification model (Figures 4 and 5). MARS is a nonparametric regression procedure that creates models based on piecewise linear regressions. It searches through all predictors to find those most useful for predicting outcomes and then creates an optimal model by a series of regression splines called basis functions [36, 37]. For this, MARS uses a two-stage process; the first half of the process involves creating an overly large model by adding basis functions that represent either single variable transformations or multivariate interaction terms. In the second stage, MARS successively deletes basis functions, starting with the lowest contributor in order of least contribution to the model until the optimum model is reached. The end result is a classification model based on single variables and interaction terms that will optimally determine class identity [36, 37].

Inputs to the model were the log2-transformed values for protein spots that were differentially abundant (31 spots) or S-NO-modified (42 spots) in HF subjects with respect to NH controls at *p* < 0.001 with B-H correction. We assessed the model accuracy using the prediction success rate and the ROC curves. The CV and 80/20 approaches identified 12 and 8 protein spots, respectively, with high importance (score >20) for creating the MARS model that permits detecting differences in abundance between the NH controls and the HF subjects (Figures 4(a) and 4(b)). The prediction success showed that the CV and 80/20 models fitted perfectly (AUC/ROC: 1.00) on the training dataset. On the testing dataset, the CV model exhibited higher prediction efficiency (AUC/ROC: 0.917) than the 80/20 model (AUC/ROC: 0.828), as is shown in Figures 4(c) and 4(d).

Likewise, the CV and 80/20 approaches identified 5 and 6 protein spots, respectively, detecting differences in S-NO modification between NH controls and the HF subjects with high importance (score >20) for creating the MARS model (Figures 5(a) and 5(b)). The prediction success showed the CV and 80/20 models fitted perfectly on the training dataset (AUC/ROC: 1.00) and by >75% on the testing dataset (AUC/ROC: 0.75 for CV and 0.857 for 80/20) (Figures 5(c) and 5(d)). These analyses suggested that PBMC changes in abundance and S-NO modification of the selected protein spots will have high specificity and sensitivity in predicting the risk of HF.

### 3.4. Verification of BD-Labeling/2D-GE Results

**Figure 6:**
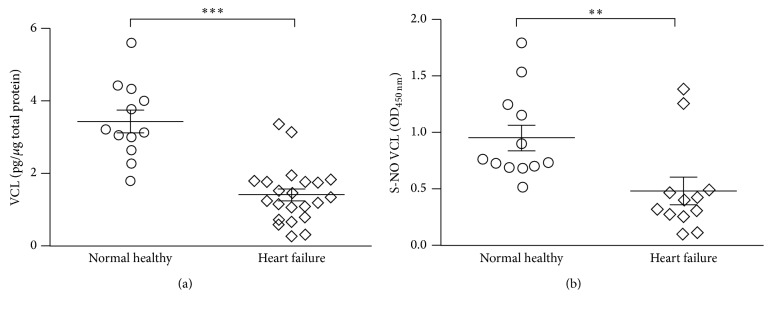
Validation of expression profile of vinculin (VCL) in HF subjects. PBMC protein lysates (5 *µ*g) from NH controls (*n* = 12) and HF subjects (*n* = 22) were subjected to sandwich and biotin-switch ELISA, respectively, for the detection of VCL (a) and SNO-modified VCL (b) levels. Mann Whitney *U* test was performed to evaluate the significance (^*∗∗*^ *p* < 0.01, ^*∗∗∗*^ *p* < 0.001).

**Figure 7:**
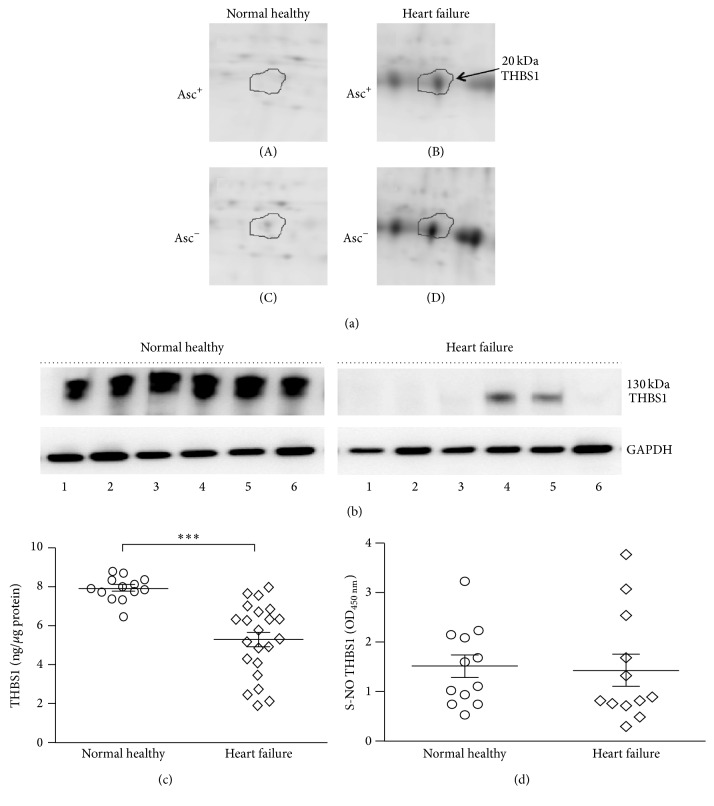
Validation of expression profile of thrombospondin 1 (THBS1) in HF subjects. (a) The expanded view of the corresponding spot for THBS1 peptides (20 kDa) from representative 2D gel images of Asc^+^ ((A) and (B)) and Asc^−^ ((C) and (D)) PBMCs from NH controls ((A) and (C)) and HF ((B) and (D)) subjects is shown. (b) PBMC lysates (5 *µ*g) of NH controls (*n* = 6) and HF subjects (*n* = 6) were subjected to Western blotting for the detection of THBS1 levels. GAPDH in (b) is shown as loading control. (c) ELISA was performed on 5 *µ*g of PBMC lysates for the detection of THBS1 abundance (NH *n* = 13, HF *n* = 22) and S-NO modification status (NH *n* = 12 and HF *n* = 12). Mann Whitney *U* test was performed to evaluate the significance ^*∗∗∗*^ *p* < 0.001. (d) Shown are the SNO-modified THBS1 levels in PBMC lysates of NH and HF subjects, determined by a biotin-switch ELISA.

Changes in abundance of four proteins, ACTB, ATP5B, VCL, and THBS1, were predictive of the risk of HF with high efficacy by IPA analysis as well as the CV and 80/20 MARS models; these proteins were also noted to be differentially S-NO modified in HF subjects (Table 1). We utilized a different set of PBMC samples from NH and HF subjects (*n* ≥ 15/group) and employed ELISA/Western blotting and biotin-switch assay, respectively, to verify the changes in abundance and S-NO levels of two proteins in HF subjects (Figures 6 and 7).

Human vinculin is a 117 kDa protein. Our BD/2D-GE approach had identified six VCL polypeptides (spot # 52, 54, 57, 58, 59, and 63; pI: 6.39–7.53) that were close to full-length protein in size (MW: 99–108 kDa) and decreased in abundance as well as S-NO modification levels in HF subjects (Table 1). ELISA and biotin-switch/ELISA showed 2.4-fold and 49% decline in VCL abundance (Figure 6(a), *p* < 0.001) and S-NO modification level (Figure 6(b), *p* < 0.01), respectively, in PBMCs of HF subjects when compared to that noted in NH controls. These data confirmed that our approach of BD-labeling/2D-GE provided a sensitive measure of proteomic changes in HF.

Human thrombospondin 1 is a 130 kDa protein. Our BD/2D-GE approach had identified five THBS1 polypeptide fragments (spot # 400, 491, 505, 509, and 732; pI: 4.72–8.72) that were increased in abundance and S-NO modification levels in HF subjects (Table 1). The 2D-GE image of one of the five THBS1 spots from representative Asc^+^ and Asc^−^ NH (Figure 7(a), panels (A) and (C)) and HF (Figure 7(a), panels (B) and (D)) PBMCs is shown. Thus, the BD/2D-GE data suggested that fragmented THBS1 was increased in abundance and S-NO modification in HF subjects. The representative Western blotting data for the 130 kDa THBS1 molecule in PBMCs of HF subjects versus NH controls showed that the intact THBS1 was indeed absent or present at very low concentrations in HF subjects (Figure 7(b)). The sandwich ELISA using a combination of antibodies that preferably detect 130 kDa THBS1 showed that the full-length THBS1 was significantly decreased in PBMCs of HF subjects (Figure 7(c), *p* < 0.001). These data, thus, confirmed that THBS1 was fragmented (as noted by 2D-GE) in HF subjects.

Because biotin-switch assay was coupled with sandwich ELISA for full-length THBS1, we did not detect the differences in S-NO levels of fragmented THBS1 between NH versus HF subjects (Figure 7(d)). An ELISA specific for the fragmented THBS1 polypeptide will be useful to distinguish the abundance and S-NO levels of functional versus cleaved THBS1 in the PBMCs of patients to predict the risk of heart failure.

## 4. Discussion

In the present study, we have performed a high throughput proteomic analysis of PBMCs from 30 heart failure patients in comparison with 30 healthy subjects. We ran 120 2D gels toresolve the protein samples and utilized BODIPY FL* N*-(2-aminoethyl) maleimide labeling as a novel method to detect changes in abundance and S-NO modification in PBMC samples. Of the 635 protein spots that were detected on 2D-gels, 93 and 111 protein spots (|≥1.5-fold|, *p* < 0.05) were found to be consistently differentially abundant (range 3-fold to −3.6-fold) or S-NOmodified (range: 3.76-fold to −5.05-fold) in HF subjects, and these protein spots were identified by MALDI-TOF MS analysis (Table 1). The finding that many of the differentially abundant protein spots were S-NO modified suggested that S-NO serves as a mechanism for regulating protein function and turnover in HF.

As the NIH/NIAID-funded Clinical Proteomics Center for Infectious Diseases and the NIH/NHLBI-funded Proteomics Center in the US, we have used the BODIPY FL-mal saturation fluorescence method for differential proteomic analysis of >2000 protein samples of diverse origin and found the assay to yield highly reproducible and quantitative results. Our protocol involved quantitation of cysteine content in samples, ensuring that the dye to thiol ratio exceeds 50 : 1 to achieve saturation dye concentration [17, 18]. Further, by using a panel of proteins including yeast enolase, bovine alpha-lactalbumin A, bovine carbonic anhydrase II, and horse myoglobulin that consist of 1, 8, 0, and 0 cysteine residues, respectively, we showed that BODIPY FL-mal (a) efficiently labels proteins in presence of thiourea at pH 7.2–8.0, and (b) dye-binding is highly specific within dye to thiol concentration ratio in the range if 5–200 : 1 and sensitive with detection limit of 0.6 fmol per cysteine in a protein spot. No detectable binding was observed with proteins containing no cysteine. Moreover, the BD labeling was linear over 4 orders of magnitude of concentration with high reproducibility in replicates (CoV: 1.9–9.4% for replicates and multiple test runs) and did not interfere with mobility of the proteins on gels and identification by mass spectrometry [18]. The protocol also employed precautions used by others in the literature, namely, neocuproine added where appropriate, sample prep (before covalent modification, locking in S-NO status) in the dark and cold, and minimal time before S-NO reduction and covalent modification of Cys-SH. The latter precaution is an improvement over the conventional approaches. Importantly, like biotin-switch assay, BODIPY FL-Mal utilizes sulfhydryl-based chemistry, requiring the thiolate ion for reaction, and is, therefore, acutely sensitive to the pH during alkylation. We precisely control the pH of the alkylation reaction. Thus, we surmise that the BD FL-mal labeling provides a powerful approach in quantification of changes in protein abundance and cysteinyl-S-nitrosylation in a variety of complex protein samples in diverse disease processes.

IPA analysis of the differentially abundant proteome dataset at the disease and functionallevel suggested the activation of phagocyte chemotaxis/migration and free radical production alongside a decrease in fatty acid metabolism (↑ACTB, ↓ALB, ↑APOA1, ↑ANXA1, ↑CPTA1, ↓GSN, ↓HBB, ↓HSPA8, ↑LTF, ↑PRDX6, ↑S100A8, and ↑THBS1) and an increase in platelet aggregation (↓ALB, ↑FGA, ↓FGB, ↓GSN, ↑RAP1B, and ↑THBS1) with HF development (Figure S1). Disease and functional network analysis of the differential abundance proteome dataset also suggested increased cell death response in HF patients (Figure S2). Interestingly, several of the proteins involved in phagocytes' migration (e.g., ANXA1, THBS1, and S100A8), cell death (e.g., YY1, RALB, RAP1B, and LTF), fatty acid metabolism (e.g., CPT1A, PRDX6, LTF, and APOA1), or leukocyte/neutrophil activation were increased in abundance as well as in S-NO levels. How increased S-NO modification contributes to activation (phagocyte migration and cell death) and downregulation (fatty acid metabolism) of specific pathways is not known. Nevertheless, our observations allow us to propose that Cys-S-nitrosylation serves an important function in regulating immune responses and cell survival in HF development. Further studies will delineate the processes of selective targeting of proteins for S-NO modification and its effect on functional activity of the proteins. It is intriguing to note that (a) phagocytes activation and a decline in fatty acid metabolism where both of which were not spared from S-NO modification were differentially regulated in HF; (b) recent studies have indicated that upregulation of glucose metabolism promotes proliferation and activation of proinflammatory macrophages [38]. Our findings provide the first indication that a metabolic shift potentially contributes to proinflammatory state in progressive HF, to be verified in future studies.

Others have shown that S-NO formation results in a decline in the bioavailability of nitric oxide required for intracellular Ca^2+^ flux, the myofilament response to Ca^2+^, and thereby can influence the systolic and diastolic performance of the myocardium [39]. It is suggested that NO/cGMP-dependent cardiac homeostasis is adversely influenced by phosphodiesterase 5 (PDE5) in the hypertrophied heart [40–42]; S-NO of PDE5 promotes its degradation by ubiquitination pathway [42]. Thus, S-NO may have beneficial as well as harmful role in the context of HF development.

MARS modeling by two different approaches indicated 14 abundant and 9 S-NO modified protein spots in the PBMC dataset to be predictive of HF development. Of these, ACTB, ATP5B, THBS1, and VCL were identified to be differentially expressed by both CV and 80/20 MARS models with high predictive efficacy. We validated the relative change in abundance of VCL and THBS1 in a second set of PBMCs from HF patients, and these proteins merit further discussion in the context of HF. Thrombospondin family consists of multimeric, multidomain calcium-binding glycoproteins that act as regulators of cell-matrix associations as well as interacting with other ECM molecules affecting their function [43]. The expression of THBS1 (and -2 and -4 isoforms) was increased in hypertensive heart disease [43]. Studies using a murine model of THBS1 genetic deletion suggested that, in cardiac remodeling after myocardial infarction, THBS1 limits the infarct expansion of the noninfarcted myocardium [44] and these benefits were delivered via activation of TGF-*β*, MMP inhibition, and CD47-mediated anti-inflammatory actions [45]. THBS2 and THBS4 isoforms have been shown to protect ECM adverse remodeling in ageing heart [46] and viral myocarditis induced HF [47]. Others have shown that THBS1 peptide antagonist prevented the progression of cardiac fibrosis and improved cardiac function by reducing TGF-*β* activity in a rat model of diabetic cardiomyopathy [48]. Our observation of increased abundance of 11–26 kDa fragments of THBS1 that were also S-NO modified in HF patients (Figure 7) suggested that THBS1 expression and catabolism was enhanced in HF patients. The finding of a significant decrease in full-length THBS1 (Figures 7(b) and 7(c)) was also in agreement with the observation of high rate of degradation and secretion of THBS1 during heart failure [49, 50]. A direct relationship between THBS1 secretion and increase in inflammation in aortic aneurysm is also noted [51]. These observations emphasize the context-dependent functions of THBSs in signaling TGF-*β* activation versus cardiac hypertrophy and heart failure. Yet, our observations of an increase in the abundance of several indicators of inflammation (discussed above), cardiac arteriopathy (↑APOA1, ↑FGA, ↑THBS1, ↑TKT, ↓TUBB1, *p* value 1.02*E* − 03), and hypertrophy (↑CPT1A, ↓GSN, ↑MYL9, ↑S100A6, ↓TPM1, *p* value 4.49*E* − 03), many of which (e.g., CPT1A, GRB2, HSPA1A/HSPA1B, and S100A6) were also increased in S-NO levels (*p* value 1.01*E* − 03) in HF subjects, suggested that fragmented THBS1 is potentially a signaling molecule in cardiac remodeling/inflammatory processes in heart failure, to be validated in future studies.

Vinculin, among others, is a component of subsarcolemmal structures, also known as costameres, in striated muscle that circumferentially align with the Z-disk of the myofibrils, and functions to allow muscle adhesion to the ECM [52]. A splice variant of VCL (termed metavinculin, MVCL) is expressed in muscle and platelets. Several studies using cellular models (e.g., cardiomyocytes, fibroblasts) have indicated that VCL/MVCL mechanically couple the actin-based cytoskeleton to the sarcolemma and regulate focal adhesion turnover [53]. Our finding of a decline in VCL in PBMCs of HF patients is in alignment with the observations made in genetically modified mice depleted of VCL expression. The VCL^−/−^ mice displayed thin-walled myocardium [54] while VCL^+/−^ mice exhibited severe pressure overload induced hypertrophy and progressive LV dysfunction associated with abnormalities of Z-line structure in the myocardium [55]. The cardiac-specific VCL knockdown resulted in early development of ventricular tachycardia followed by cardiomyopathy and all mice died before 6 months of age [56]. The absence of cardiac VCL was associated with highly serrated intercalated disks that connect myocytes end-to-end and loosely arranged and disorganized mitochondria in cardiomyocytes [56]. Others have reported linkage of multiple mutations of VCL with dilated and hypertrophic forms of human cardiomyopathy [57, 58]. These studies show a clear linkage in alteration of VCL expression with cardiomyopathy and heart failure, emphasize the critical role of VCL/MVCL in maintenance of cardiac function, and provide us with impetus to evaluate peripheral VCL levels as a risk factor in heart failure development.

In summary, current study was focused on the S-NO modification and its impact on the pathophysiology in HF. Future studies will be required to investigate the interplay between protein nitrosylation and other cysteine-based PTM in heart failure. A number of other posttranslational modifications (PTMs), for example, acetylation,* O‐*GlcNAcylation, 3-nitrotyrosine, and carbonyls, have also been implicated in HF of diverse etiologies [59]. The pathophysiological significance of PTMs with a potential impact on protein misfolding, function, and ultimate disease outcomes in HF is recently reviewed in excellent review article [60]. Our proteome analysis of the PBMCs from HF patients showed differential abundance and S-NO modification of proteins involved in cell viability and production of reactive oxygen species and indicated the potential of THBS1 and VCL evaluation to be useful in the prediction for risk of heart failure.

## Supplementary Material

The proteome datasets were submitted to Ingenuity Pathway Analysis for identifying the networks and signaling pathways that were potentially disturbed in heart failure subjects. The differentially abundant proteins involved in disease and disorder network of inflammation (Figure S1), and disease and bio-function network of increased cell death and decreased cell survival (Figure S2) in heart failure (HF) subjects are presented. Further, the differentially S-NO-modified proteins involved in migration of phagocytes and inhibition of cell spreading (Figure S3) and in organismal death and cell apoptosis with production of free radicals (Figure S4) in HF subjects are presented.

## Figures and Tables

**Figure 2:**
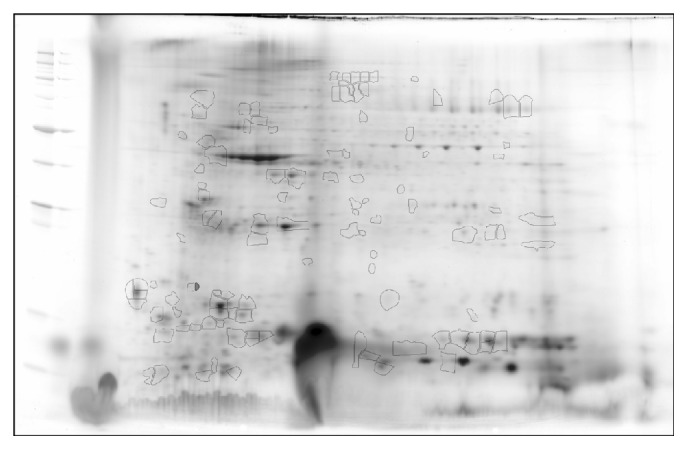
Identification of differentially abundant and S-NO-modified protein spots in PBMCs of HF subjects. Protein spots that exhibited significant change in abundance or S-NO-modification in HF subjects with respect to NH controls (*p* < 0.05) are marked on the reference gel and were submitted for protein identification by MALDI-TOF MS analysis (listed in Table 1).

**Table 1:** Changes in abundance and S-nitrosylation proteome profile of PBMC proteins in human heart failure.

2D-GE data			MALDI MS data				^2^ASC^−^/ASC^+^ (ΔSNO) ratio		^1^Abundance (ASC^+^)	^3^S-NO ROR
Spot number	pI	MW (kD)	Plate number	Protein and gene name	Accession number	MS (LC MS/MS) ID protein score	NH	HF	HF versus NH	HF versus NH
52	6.39	108	F1	Vinculin GN=VCL	P18206	709^*∗*^	1.14	−1.03	−1.88	1.83
54	6.57	108	H14	Vinculin GN=VCL	P18206-2	1180^*∗*^	1.08	1.09	−3.29	3.58
57	6.68	107	H15	Vinculin GN=VCL	P18206-2	1130^*∗*^	−1.03	−1.06	−3.09	2.91
58	6.80	107	F20	Vinculin GN=VCL	P18206-2	1210^*∗*^	−1.01	1.04	−3.61	3.76
59	6.94	107	F21	Vinculin GN=VCL	P18206	1170^*∗*^	1.01	1.04	−3.27	3.39
60	7.09	105	C14	FAM212B GN=FAM212B	Q9NTI7	29	1.04	−1.06	−2.38	2.25
63	7.53	99	7	Vinculin GN=VCL	P18206	236^*∗*^	1.12	−1.24	−1.54	1.24
79	6.48	96	F22	Albumin GN=ALB	P02768	190^*∗*^	−1.28	−1.14	−2.03	1.78
81	6.62	94	F23	Keratin, type I cytoskeletal 9 GN=KRT9	P35527	188^*∗*^	−1.18	−1.23	−2.37	1.93
92	6.93	87	F24	Filamin-A GN=FLNA	Q5HY54	384^*∗*^	−1.17	−1.56	−1.20	−1.31
95	6.81	87	G2	Filamin-A GN=FLNA	Q5HY54	399^*∗*^	−1.19	−1.22	−2.02	1.66
99	4.54	84	G4	Glucose-regulated 78 kDa protein GN=HSPA5	P11021	849^*∗*^	1.01	1.29	−1.61	2.08
102	6.68	83	G5	Filamin-A GN=FLNA	Q5HY54	210^*∗*^	−1.26	−1.32	−1.64	1.24
103	6.41	82	H21	Filamin-A GN=FLNA	P21333	238^*∗*^	−1.28	−1.03	−1.68	1.63
104	6.52	82	I20	Gelsolin GN=GSN	P06396-2	182^*∗*^	−1.32	−1.11	−1.72	1.55
124	5.21	68	G6	Heat shock 71 kDa protein GN=HSPA8	P11142-2	325^*∗*^	1.04	−1.01	−1.52	1.51
127	9.09	68	G7	Fibrinogen alpha chain GN=FGA	P02671-2	62^*∗*^	1.07	−1.65	1.54	−2.53
131	5.35	68	G8	Keratin, type I cytoskeletal 10 GN=KRT10	P13645	296^*∗*^	−1.15	−1.08	−1.72	1.59
136	8.84	68	E6	Transketolase GN=TKT	B4E022	189^*∗*^	−1.03	−1.88	1.84	−3.46
141	5.97	63	1	Serum albumin GN=ALB	H0YA55	167^*∗*^	−1.14	1.09	1.07	1.03
142	4.51	63	G9	Tropomyosin 3 GN=TPM3	Q5VU59	743^*∗*^	1.01	1.34	−1.83	2.46
145	6.41	60	A1	Serum albumin GN=ALB	H0YA55	245^*∗*^	−1.59	−1.28	−1.04	−1.24
146	6.78	60	C15	Serum albumin GN=ALB	P02768	116^*∗*^	−1.00	−1.42	1.32	−1.88
154	5.34	59	H24	Actin, cytoplasmic 1 GN=ACTB	P60709	391^*∗*^	−1.43	−1.07	−1.55	1.44
180	5.14	54	J11	Actin, cytoplasmic 1 GN=ACTB	P60709	353^*∗*^	−1.14	1.18	−1.76	2.07
185	5.50	53	K19	Actin, cytoplasmic 1 GN=ACTB	P60709	365^*∗*^	1.28	−1.16	1.41	−1.63
197	7.48	51	G12	Fibrinogen beta chain GN=FGB	P02675	157^*∗*^	−1.14	−1.07	−1.53	1.43
204	4.29	50	G13	Nucleosome assembly protein 1 GN=NAP1L1	F8VRJ2	79^*∗*^	1.21	−1.07	−1.74	1.62
205	8.50	50	C16	Fibrinogen beta chain GN=FGB	P02675	336^*∗*^	−1.11	−1.18	1.00	−1.18
222	4.71	47	J13	ATP synthase subunit beta GN=ATP5B	H0YH81	302^*∗*^	−1.06	1.20	−1.92	2.30
241	8.79	45	J1	Transcription factor 4 GN=TCF4 (integrin-linked protein kinase GN=ILK)	H3BME8	36 (305)^*∗*^	−1.73	−1.16	−1.68	1.45
261	6.57	41	101	POTE ankyrin domain family member F	A5A3E0	121^*∗*^	−1.47	1.16	−1.35	1.57
262	7.68	41	I5	Elongation factor Tu, mitochondrial GN=TUFM	P49411	195^*∗*^	−1.52	−1.04	−1.58	1.52
264	4.86	41	J18	Actin, cytoplasmic 1 GN=ACTB	P60709	336^*∗*^	−1.05	1.06	−1.49	1.58
267	6.41	41	16	Actin, cytoplasmic 2, N-terminal	F5H0N0	106^*∗*^	−1.19	1.33	−1.15	1.53
272	8.70	41	B9	Myeloperoxidase GN=MPO	P05164-2	83^*∗*^	−1.30	−1.47	1.22	−1.79
303	4.54	38	B10	Actin, cytoplasmic 1 GN=ACTB	P60709	91^*∗*^	1.17	1.18	−1.32	1.55
329	7.17	35	I10	DnaJ homolog subfamily B 11 GN=DNAJB11	H7C2Y5	41	−1.25	−1.46	1.17	−1.70
335	5.55	35	D6	Actin, cytoplasmic 1 GN=ACTB	P60709	225^*∗*^	−1.14	−1.09	1.88	−2.04
337	5.81	34	D7	Actin, cytoplasmic 1 GN=ACTB	P60709	456^*∗*^	−1.20	−1.06	1.64	−1.74
339	6.33	34	105	Tubulin beta chain GN=TUBB	Q5JP53	121^*∗*^	−1.28	−1.38	1.19	−1.64
343	6.72	34	B13	Actin, cytoplasmic 1 GN=ACTB	P60709	97^*∗*^	−1.17	−1.36	1.26	−1.71
344	3.63	33	A2	Stromal interaction molecule 2 GN=STIM2	Q9P246	33	1.43	−1.18	1.98	−2.34
348	7.11	33	D8	Phosphoglycerate kinase GN=PGK1	B7Z7A9	54	−1.23	−1.92	1.49	−2.86
358	4.56	31	G24	Tropomyosin 1 alpha isoform 7 GN=TPM1	D9YZV8	515^*∗*^	1.20	1.13	−1.52	1.72
373	7.32	30	H1	Annexin A1 GN=ANXA1	P04083	200^*∗*^	−1.18	−2.15	−1.13	−1.91
384	4.02	29	L10	LVV-hemorphin-7 GN=HBB	F8W6P5	47	−1.09	1.57	−1.98	3.12
385	5.72	29	102	Vimentin GN=VIM	F5H288	171^*∗*^	−1.22	−1.36	1.20	−1.63
395	6.85	27	E8	Keratin, type II cytoskeletal 2 GN=KRT2	P35908	381^*∗*^	−1.46	−1.65	1.70	−2.80
397	4.01	27	J23	Tropomyosin alpha-4 chain GN=TPM4	P67936	101^*∗*^	1.07	1.41	−1.58	2.22
400	6.70	26	L2	Thrombospondin-1 GN=THBS1	P07996	769^*∗*^	−1.14	1.05	2.00	−1.91
404	7.51	26	17	Actin, cytoplasmic 1, N-terminal GN=ACTB	B4DW52	133^*∗*^	1.23	−1.09	1.91	−2.08
406	8.08	26	K1	Uncharacterized protein FLJ46347	Q6ZRH9	36	−1.07	−1.16	1.88	−2.18
411	7.98	26	5	Myosin-IXa GN=MYO9A	H3BMM1	46	1.09	−1.27	2.26	−2.86
424	4.69	25	F15	Tropomyosin alpha-4 chain GN=TPM4	P67936	926^*∗*^	1.73	1.18	1.85	−1.57
425	8.63	25	12	Peptidyl-prolyl cis-trans isomerase A GN=PPIA	P62937	110^*∗*^	−1.36	−1.17	1.46	−1.71
431	6.70	24	H3	Haloacid dehalogenase-like hydrolase domain-containing protein 2 GN=HDHD2	K7ER15	102^*∗*^	−1.20	−1.27	1.42	−1.80
451	9.33	23	F16	Glyceraldehyde-3-phosphate dehydrogenase GN=GAPDH	E7EUT4	71^*∗*^	1.02	−1.36	1.12	−1.52
461	6.98	22	B17	Annexin A1 GN=ANXA1	P04083	344^*∗*^	1.23	−1.07	1.55	−1.65
476	4.74	21	D10	Actin, cytoplasmic 1 GN=ACTB	P60709	226^*∗*^	1.05	1.22	1.51	−1.24
491	8.58	20	B18	Thrombospondin-1 GN=THBS1	P07996	138^*∗*^	1.20	−1.20	2.45	−2.95
501	6.78	20	H6	Peroxiredoxin-6 GN=PRDX6	P30041	237^*∗*^	−1.08	−1.48	1.51	−2.23
505	8.22	20	I14	Thrombospondin-1 GN=THBS1	P07996	197^*∗*^	1.06	1.07	1.52	−1.42
507	6.61	20	H7	Growth factor receptor-bound protein 2 GN=GRB2	P62993-2	80^*∗*^	−1.01	−1.14	1.48	−1.68
509	8.72	20	L3	Thrombospondin-1 GN=THBS1	P07996	418^*∗*^	−1.03	−1.29	2.74	−3.53
511	7.30	20	D14	Carnitine O-palmitoyltransferase 1 GN=CPT1A	P50416	32	−1.15	−1.03	1.65	−1.70
514	4.10	20	E9	Transcriptional repressor protein YY1 GN=YY1	H0YJV7	47	1.01	1.27	−1.43	1.82
515	5.26	19	L4	Apolipoprotein A-I GN=APOA1	P02647	352^*∗*^	1.06	1.11	1.56	−1.40
518	4.29	19	C21	Vimentin GN=VIM	B0YJC4	203^*∗*^	1.13	1.37	−1.17	1.60
524	9.34	19	108	Keratin, type I cytoskeletal 10 GN=KRT10	P13645	286^*∗*^	−1.02	−1.47	1.42	−2.08
530	8.61	19	L11	Keratin, type II cytoskeletal 1 GN=KRT1	P04264	55	1.42	−1.35	3.00	−4.04
549	6.94	19	J5	Glutathione GN=GSH (Ras-related protein Rab-14 GN=RAB14)	P07203	71 (288)^*∗*^	1.10	−1.13	1.57	−1.78
563	5.99	18	109	Actin, cytoplasmic 2, N-terminal GN=ACTB	I3L1U9	137^*∗*^	−1.53	1.07	−1.60	1.71
567	6.93	18	C22	Glutathione S-transferase P GN=GSTP1	P09211	286^*∗*^	−1.02	−1.30	1.26	−1.64
568	3.62	18	E10	Transcriptional repressor protein YY1 GN=YY1	H0YJV7	39	1.47	−1.22	1.83	−2.24
574	4.65	17	C23	Tubulin beta-1 chain GN=TUBB1	Q9H4B7	39	1.21	1.28	−2.09	2.66
588	3.88	17	A8	Alpha-actinin-1 GN=ACTN1 (Alpha actinin 1 GN=ACTN1)	H0YJ11	50 (222)^*∗*^	1.44	1.24	−1.28	1.59
591	7.55	17	E12	Heat shock 70 kDa protein 1A/1B GN=HSPA1A	E7EP11	43	1.01	−1.07	1.43	−1.52
592	4.38	17	106	Myosin regulatory light chain 12B GN=MYL12B	O14950	320^*∗*^	1.43	1.62	−1.71	2.78
593	4.47	17	C24	Actin, cytoplasmic 1 GN=ACTB	P60709	65^*∗*^	1.63	1.75	−1.66	2.91
595	8.27	17	A9	Protein S100-A8 GN=S100A8	P05109	60	1.59	−1.07	1.65	−1.76
603	4.76	16	A10	Annexin A5 GN=ANXA5	D6RBL5	111^*∗*^	−1.20	−1.37	1.13	−1.55
605	5.64	16	110	ATP synthase subunit alpha GN=ATP5A1	A8K092	95^*∗*^	−1.03	−1.00	−1.02	1.01
607	6.71	16	B20	Actin-related protein 2/3 complex subunit 3 GN=ARPC3	O15145	50	1.06	1.14	1.64	−1.43
612	4.19	16	K21	Myosin regulatory light chain 9 GN=MYL9	P24844	233^*∗*^	1.19	1.38	1.62	−1.17
613	4.61	16	D17	Actin, cytoplasmic 2 GN=ACTG1	P63261	61	1.45	1.21	1.78	−1.47
623	5.16	16	A12	Actin, cytoplasmic 2, N-terminal GN=ACTG1	K7EM38	130^*∗*^	1.30	1.29	1.87	−1.45
627	5.47	15	3	Annexin A6 GN=ANXA6	E5RIU8	103^*∗*^	−1.25	1.03	1.30	−1.26
628	3.79	15	E14	Myosin light polypeptide 6 GN=MYL6	F8VZV5	302^*∗*^	1.30	1.32	1.12	1.18
629	4.35	15	A13	Actin, cytoplasmic 1 GN=ACTB	P60709	65^*∗*^	1.24	1.32	1.15	1.14
630	8.05	15	K6	Peptidyl-prolyl cis-trans isomerase A GN=PPIA	P62937	199^*∗*^	−1.04	−1.18	−1.10	−1.07
632	9.02	15	I15	Peptidyl-prolyl cis-trans isomerase A GN=PPIA	P62937	235^*∗*^	−1.01	−1.12	−1.20	1.07
640	5.12	15	13	Actin, cytoplasmic 1, N-terminal GN=ACTB	G5E9R0	193^*∗*^	1.45	1.40	1.82	−1.30
642	4.21	15	E15	Actin, cytoplasmic 1 GN=ACTB	P60709	139^*∗*^	1.05	1.25	−1.31	1.64
644	7.71	15	21	Keratin, type I cytoskeletal 10 GN=KRT10	P13645	47	1.16	1.32	−1.07	1.41
646	3.98	15	H9	Myosin light polypeptide 6 GN=MYL6	F8VZV5	369^*∗*^	1.16	1.41	−1.39	1.97
648	8.37	15	D1	Bestrophin-3 GN=BEST3 (dermcidin GN=DCD)	A8MVM3	42 (160)^*∗*^	1.17	1.98	1.29	1.53
657	4.79	15	A14	Actin, cytoplasmic 1 GN=ACTB	P60709	108^*∗*^	1.25	1.12	1.62	−1.44
662	4.65	14	A16	Actin, cytoplasmic 1 GN=ACTB	P60709	263^*∗*^	1.55	1.62	1.65	−1.02
664	7.01	14	D19	Actin, cytoplasmic 2, N-terminal GN=ACTG1	K7EM38	41	1.53	1.09	1.29	−1.18
665	4.45	14	A17	Actin, cytoplasmic 1 GN=ACTB	P60709	233^*∗*^	1.56	1.16	1.51	−1.30
666	3.82	14	B22	Mitochondrial carrier homolog 1 GN=MTCH1	Q9NZJ7-3	32	1.34	1.40	−1.01	1.42
671	8.66	14	B23	Bestrophin-3 GN=BEST3	A8MVM3	40	1.21	1.43	2.00	−1.40
673	4.03	14	B24	Isoform H14 of Myeloperoxidase GN=MPO	P05164-2	288^*∗*^	−1.16	1.18	−1.48	1.76
683	5.01	13	A22	Histone H4 GN=HIST1H4A	P62805	242^*∗*^	1.01	−1.09	1.44	−1.56
690	7.92	13	L13	Hemoglobin subunit beta GN=HBB	P68871	384^*∗*^	−1.15	−1.12	1.50	−1.68
703	7.72	12	D3	LVV-hemorphin-7 (fragment) GN=HBB	F8W6P5	41	−1.13	1.09	−1.49	1.63
706	6.70	12	E16	Protein S100-A11 GN=S100A11	P31949	260^*∗*^	−1.39	1.27	−1.75	2.22
718	4.53	11	C4	Ras-related protein Ral-B GN=RALB (actin cytoplasmic 2 GN=ACTG1)	F8WEQ6	48 (155)^*∗*^	1.20	−1.30	1.64	−2.14
732	4.72	11	C6	Thrombospondin-1 GN=THBS1	P07996	115^*∗*^	1.61	−2.02	2.50	−5.05
737	4.00	10	D20	ATP synthase subunit alpha GN=ATP5A1	K7EQT2	173^*∗*^	−1.16	−1.90	2.40	−4.57
738	3.88	10	D21	Keratin, type II cytoskeletal 1 GN=KRT1	P04264	96^*∗*^	1.07	−1.46	1.85	−2.70
740	4.86	0	C7	Urea transporter 1 GN=SLC14A1	K7EJ54	39	1.33	−1.35	1.78	−2.40
744	4.40	0	23	Ras-related protein 1b GN=RAP1B	B4DQI8	58	−1.02	−1.30	1.81	−2.35
759	4.82	15	D22	Actin, cytoplasmic 2, N-terminal GN=ACTG1	K7EM38	263^*∗*^	1.27	1.37	1.73	−1.27
761	4.82	16	D23	Keratin, type II cytoskeletal 1 GN=KRT1	P04264	789^*∗*^	1.24	1.31	1.62	−1.24
762	4.91	16	L14	Actin, cytoplasmic 2 GN=ACTG1	P63261	221^*∗*^	1.34	1.18	1.69	−1.44
763	5.73	20	A24	Actin, cytoplasmic 1 GN=ACTB	P60709	416^*∗*^	−1.06	−1.09	1.89	−2.05
769	5.38	14	B1	Actin, cytoplasmic 1 GN=ACTB	C9JUM1	44	1.07	−1.05	1.59	−1.67
772	5.37	13	B2	Histone H4 GN=HIST1H4A	P62805	193^*∗*^	−1.36	−1.36	1.13	−1.54
774	3.72	16	J7	Calmodulin GN=CALM1	P62158	367^*∗*^	−1.11	1.47	−1.51	2.23
779	4.63	28	D24	Actin, cytoplasmic 1 GN=ACTB	P60709	428^*∗*^	1.16	1.21	−1.28	1.55
781	4.61	27	E1	Actin, cytoplasmic 1 GN=ACTB	P60709	342^*∗*^	1.04	1.01	−1.07	1.08
796	5.29	13	E19	Keratin, type II cytoskeletal 1 GN=KRT1	P04264	127^*∗*^	1.29	−1.70	2.35	−4.00
797	5.34	13	E20	Keratin, type II cuticular Hb3 GN=KRT83	P78385	165^*∗*^	1.08	−1.11	1.59	−1.77
804	6.96	10	E21	LVV-hemorphin-7 (fragment) GN=HBB	F8W6P5	122^*∗*^	1.26	1.36	−1.40	1.90
808	8.11	11	L6	Platelet basic protein GN=PPBP	P02775	90^*∗*^	1.43	1.19	−1.38	1.64
825	8.65	72	C8	Lactotransferrin delta GN=LTF	P02788-2	147^*∗*^	−1.44	−1.65	1.51	−2.49
854	8.74	67	C9	Kaliocin-1 GN=LTF	E7EQB2	46	−1.09	−1.50	1.22	−1.83
866	4.88	10	C10	S100-A6 protein GN=S100A6	P06703	39	1.46	−1.12	1.94	−2.18
867	4.91	0	C11	LVV-hemorphin-7 GN=HBB	F8W6P5	65^*∗*^	1.26	1.16	1.50	−1.29
868	3.59	16	I18	Calmodulin GN=CALM1	P62158	343^*∗*^	−1.01	1.27	−1.70	2.16
870	3.64	16	K10	Calmodulin GN=CALM1 (calmodulin GN=CALM1)	E7ETZ0	63 (215)^*∗*^	1.45	1.60	−1.34	2.15
871	3.69	16	K11	Calmodulin GN=CALM1	E7ETZ0	195^*∗*^	1.33	1.31	−1.49	1.95
877	3.80	55	B4	Stromal interaction molecule 2 GN=STIM2	Q9P246	39	1.31	−1.30	1.69	−2.20
879	6.87	11	E23	LVV-hemorphin-7 GN=HBB	F8W6P5	213^*∗*^	−1.02	1.20	−1.40	1.69
889	7.80	70	H12	Fibrinogen alpha chain GN=FGA	P02671-2	165^*∗*^	−1.04	−1.32	1.87	−2.48
890	4.94	16	E3	Actin, alpha 1, skeletal muscle GN=ACTA1	Q5T8M8	124^*∗*^	1.24	1.29	1.31	−1.01
891	5.03	15	E4	Actin, cytoplasmic 2, N-terminal GN=ACTG1	K7EM38	113^*∗*^	1.23	1.14	1.82	−1.59
892	7.57	13	E5	Annexin A2 GN=ANXA2	H0YKV8	109^*∗*^	−1.32	1.17	−1.49	1.75
897	4.30	14	B6	Actin, cytoplasmic 1 GN=ACTB	P60709	242^*∗*^	1.15	1.33	1.51	−1.14
901	8.64	13	L7	Hemoglobin subunit beta GN=HBB	P68871	254^*∗*^	1.17	−1.04	1.56	−1.62
902	8.60	13	L8	Hemoglobin subunit beta GN=HBB	P68871	350^*∗*^	1.02	−1.04	1.61	−1.67
903	8.60	13	L9	Hemoglobin subunit beta GN=HBB	P68871	353^*∗*^	1.18	1.02	1.95	−1.90
904	8.26	13	L15	Hemoglobin subunit beta GN=HBB	P68871	349^*∗*^	1.07	−1.02	1.72	−1.76
905	8.29	13	K23	Hemoglobin subunit beta GN=HBB	P68871	330^*∗*^	−1.09	−1.07	1.59	−1.70
911	4.69	10	I19	SH3 domain-binding glutamic acid-rich protein 3 GN=SH3BGRL3	Q9H299	354^*∗*^	1.39	−1.20	1.59	−1.91

The PBMC protein samples from normal healthy (NH, *n* = 30) and heart failure (HF, *n* = 30) subjects were incubated with (Asc^+^) or without (Asc^−^) ascorbate and resolved by 2D-GE approach. Gels were labeled with BODIPY FL *N*-(2-aminoethyl) maleimide, images were analyzed with SameSpotst*™* software, and normalized spot volumes were used for comparison. Proteins spots with ≥|1.5| fold change in abundance or S-nitrosylation level (*p* < 0.05) in HF subjects were subjected to MALDI-TOF MS/MS analysis and those identified with high confidence (score >62) are shown with *∗*. Some of the protein spots were also identified by LC MS/MS and this information is presented in brackets in protein and gene name and ID score columns.

^1^Ratiometric calculation from BODIPY-fluorescence units in Asc^+^ aliquots (normal versus experimental) was conducted for quantifying the differential abundance of protein spots (Δprotein abundance = Asc^+^ HF/Asc^+^ NH).

^2^The S-NO modification levels were quantified by calculation of the ratio of fluorescence units from Asc^−^ aliquots (ΔS-NO = Asc^−^ HF/Asc^−^ NH).

^3^The ratio of ratios, that is, ΔS-NO/Δprotein abundance = [Asc^−^ HF/Asc^−^ NH]/[Asc^+^ HF/Asc^+^ NH], was calculated to obtain the change in S-NO levels normalized for protein abundance. As S-NO modification inhibits the Cys-BODIPY fluorescence, a negative (green) and a positive (red) RoR value would indicate an increase and decrease in S-NO levels, respectively, in HF (versus NH) subjects.
