# Supplementary material for: S-Nitrosylation Proteome Profile of Peripheral Blood Mononuclear Cells in Human Heart Failure
Source: Int J Proteomics. 2016 Aug 18;2016:1384523. doi: 10.1155/2016/1384523 (PMC5007369; doi:10.1155/2016/1384523)
Supplement: Supplementary file 1 — The proteome datasets were submitted to Ingenuity Pathway Analysis for identifying the networks and signaling pathways that were potentially disturbed in heart failure subjects. The differentially abundant proteins involved in disease and disorder network of inflammation (Figure S1), and disease and bio-function network of increased cell death and decreased cell survival (Figure S2) in heart failure (HF) subjects are presented. Further, the differentially S-NO-modified proteins involved in migration of phagocytes and inhibition of cell spreading (Figure S3) and in organismal death and cell apoptosis with production of free radicals (Figure S4) in HF subjects are presented. [file 1384523.f1.pdf]

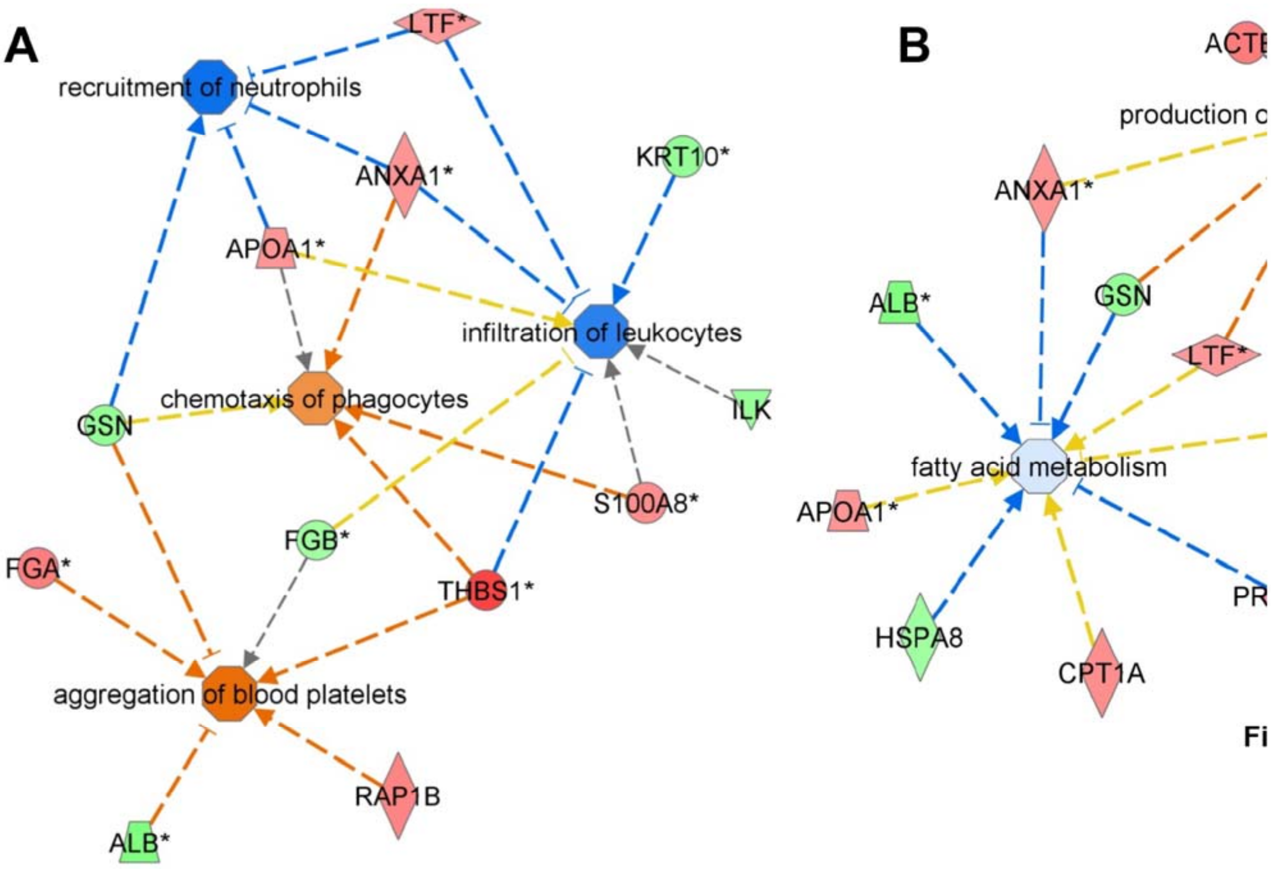

Fig.S1. PBMC proteome of heart failure (HF) subjects (versus normal healthy (NH) controls) was de Materials and Methods, and protein spots that were differentially abundant in HF subjects (fold chang identified by mass spectrometry. Shown are **(A)** disease and disorder network of inflammation in aggregation of platelets and migration of phagocytes and a decline in recruitment/infiltration of neu and **(B)** disease and bio-function network suggesting increase in ROS production and a decline in HF subjects. These networks were developed by Ingenuity pathway analysis of differential PBMC p 1). In Figs.S1&S2, the intensity of red and green colors show the extent of increase and decrease respectively, in HF individuals as compared to that noted in NH controls. Gray lines indicate putative or findings inconsistent with state of downstream molecule. Brown/orange node/lines and blue no activation and inhibition, respectively, of a pathway.

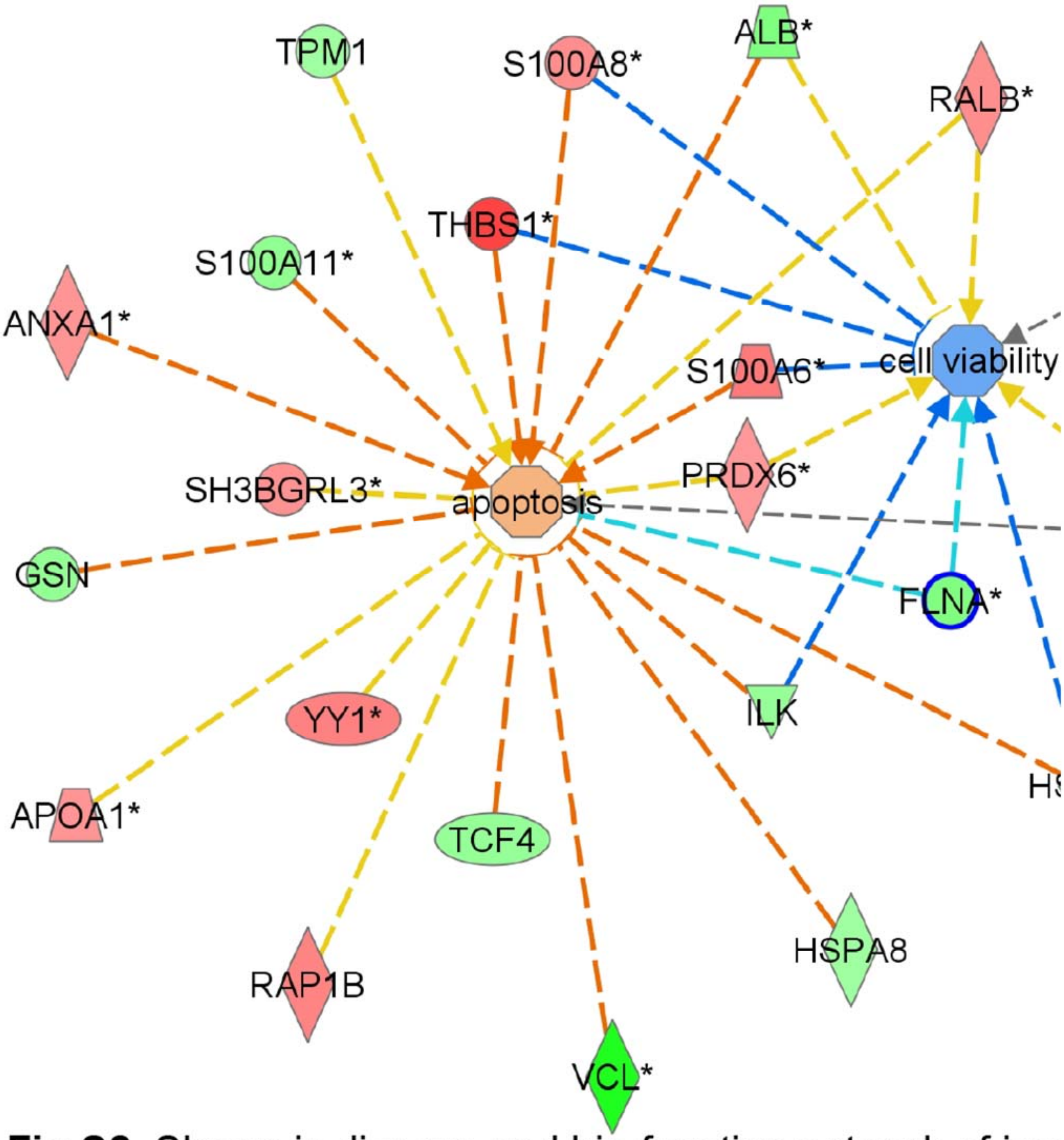

Fig.S2. Shown is disease and bio-function network of inc
death and decreased cell survival in HF subjects versus r
healthy controls; developed by Ingenuity pathway analysi
differential PBMC proteome abundance dataset (Table 1).

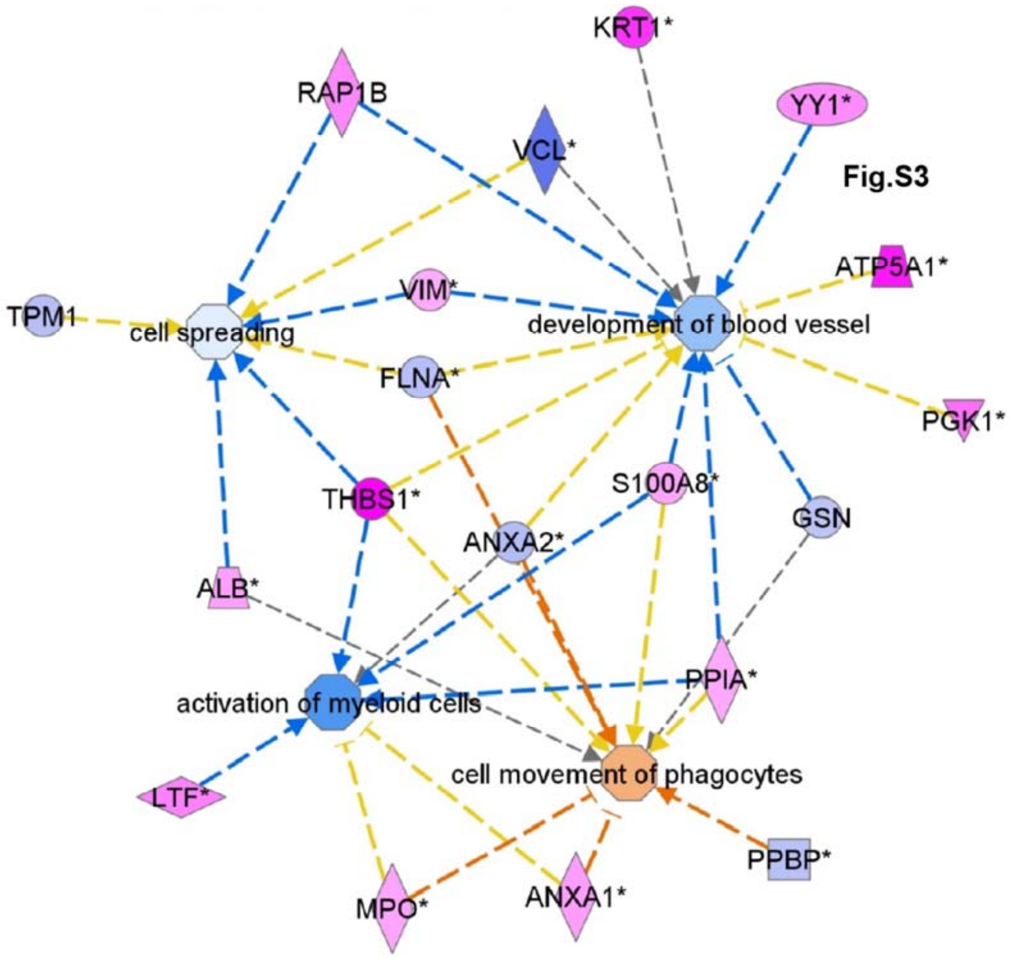

Fig.S3

Fig.S3. PBMC pr
(HF) subjects (
(NH) controls)
described in Mate
protein spots tha
NO modified
change: |≥1.5|, p<
mass spectrome
and disorder ne
increased S-nitro
involved in migra
inhibition of
development of
versus normal
pathways were
pathway analysis
PBMC proteome
Figs.S3&S4, the
and blue colors
increase and dec
respectively, in
compared to that
Gray lines indica
predicted. Brown
blue node/lines sh
and inhibition, res

Fig.S4

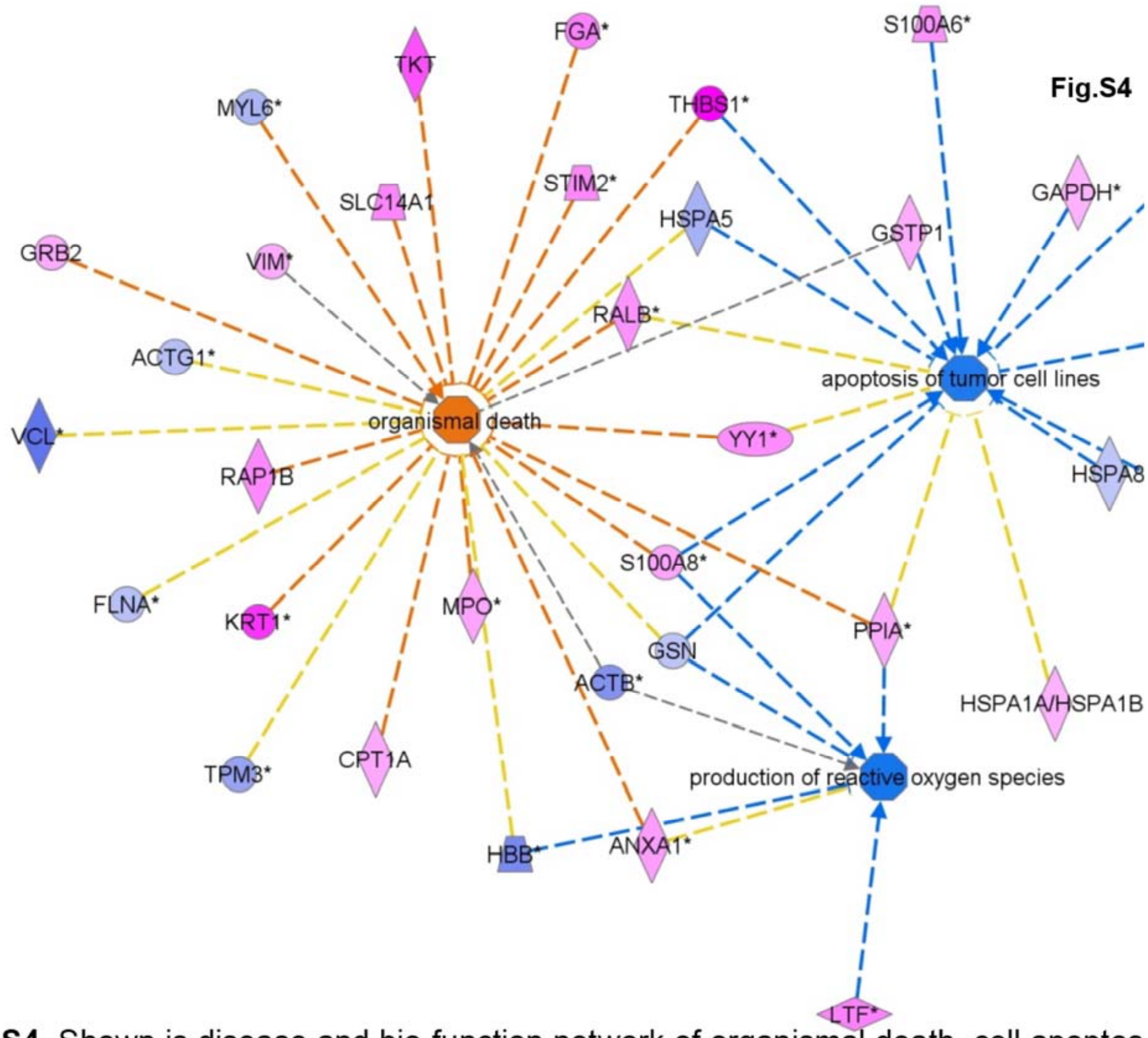

Fig.S4. Shown is disease and bio-function network of organismal death, cell apoptos of free radicals in HF subjects versus normal healthy controls; developed by In analysis of differential PBMC proteome S-NO dataset (Table 1). Note that a majority inhibit apoptosis and free radical production as well activate organismal death were suggesting that S-NO modification serves as an important mechanism in regulating ce
